# The relationship between complex PTSD and dissociation: longitudinal findings across Western and South Asian female samples

**DOI:** 10.1007/s00127-026-03053-z

**Published:** 2026-02-16

**Authors:** Hong Wang Fung, Celinene M. Lay, Guangzhe Frank Yuan, Anson Kai Chun Chau, Marc Eric S. Reyes, Edo S. Jaya, Firdaus Mukhtar, Amos En Zhe Lian, Görkem Derin, Peejay D. Bengwasan, Georgekutty Kochuchakkalackal Kuriala, Kadir Uludag, Steffi Hartanto, Nimaz Indryastuti Dewantary, Riangga Novrianto, Zoe Jiwen Zhang, Chak Hei Ocean Huang, Shao-Cheng Wang, Stanley Kam Ki Lam

**Affiliations:** 1 School of Nursing, The Hong Kong Polytechnic University, Hung Hom, Hong Kong SAR China; 2 Mental Health Research Centre, The Hong Kong Polytechnic University, Hung Hom, Hong Kong SAR China; 3 Yale School of Medicine, New Haven, Connecticut, USA; 4 School of Education Science, Leshan Normal University, Leshan, China; 5 Department of Psychiatry, The University of Hong Kong, Pok Fu Lam, Hong Kong SAR, China; 6 Department of Psychology, College of Science, University of Santo Tomas, Manila, Philippines; 7 Research Center for Social Sciences and Education, University of Santo Tomas, Manila, Philippines; 8 Faculty of Psychology, Universitas Indonesia, Depok, Indonesia; 9 Indonesian Psychological Healthcare Center, Yogyakarta, Indonesia; 10 Department of Psychiatry, Faculty of Medicine and Health Sciences, Universiti Putra, Serdang, Malaysia; 11 Faculty of Social Sciences, Raffles University, Johor Bahru, Malaysia; 12 Kepha Institute, Columbia International University, Columbia, USA; 13 Department of Social Sciences, Psychotraumatology and Psychohistory Research Unit, Istanbul University-Cerrahpaşa, Institute of Forensic Sciences and Legal Medicine, Istanbul, Turkey; 14 Department of Psychology, De La Salle University, Manila, Philippines; 15 Department of Psychology, Newman College, Thodupuzha, Kerala India; 16 School of Medicine, Shanghai Jiao Tong University, Mental Health Center, Shanghai, China; 17 Indonesian Psychological Healthcare Center, Yogyakarta, Indonesia; 18 Indonesian Psychological Healthcare Center, Jakarta, Indonesia; 19 Faculty of Psychology, Universitas Gadjah Mada, Yogyakarta, Indonesia; 20 Department of Applied Social Sciences, The Hong Kong Polytechnic University, Hung Hom, Hong Kong SAR China; 21 Department of Social Work and Social Administration, The University of Hong Kong, Pok Fu Lam, Hong Kong SAR China; 22 Department of Psychiatry, Kaohsiung Chang Gung Memorial Hospital, Chang Gung University College of Medicine, Kaohsiung, 833 Taiwan; 23 Department of Psychiatry, Kaohsiung Municipal Ta-Tung Hospital, Kaohsiung, 801 Taiwan; 24 School of Nursing and Health Sciences, Hong Kong Metropolitan University, Ho Man Tin, Hong Kong SAR, China

**Keywords:** Trauma, Complex PTSD (CPTSD), Dissociative disorders, Cross-cultural psychiatry

## Abstract

**Purpose:**

Post-traumatic stress disorder (PTSD) and dissociation are common responses to trauma, especially interpersonal and betrayal trauma. Dissociation has been proposed to be a core concept in understanding PTSD. However, little is known about the bidirectional relationship between dissociation and ICD-11 complex PTSD (CPTSD) symptoms. This study examined the relationship between classical PTSD, disturbances in self-organization (DSO), and dissociative symptoms across two culturally different samples.

**Methods:**

Participants from Western and South Asian countries completed validated measures of PTSD, DSO, and dissociation two times, approximately six months apart.

**Results:**

Across the Western (*N* = 101) and South Asian (*N* = 160) samples, at baseline, 71.7% to 84.2% of participants with probable CPTSD exhibited co-occurring dissociative symptoms, while 70.0% to 72.3% of participants with dissociative symptoms had probable PTSD or CPTSD. Dissociative symptoms were less common in participants with probable PTSD (20.0% to 28.6%). In addition, dissociative symptoms predicted subsequent levels of classical PTSD symptoms across the two samples (β = 0.241 to 0.246, *p* < .01). The predictive role of dissociative symptoms on DSO symptoms was only observed in the South Asian sample (β = 0.231, *p* = .011). Neither PTSD nor DSO symptoms predicted dissociative symptoms in both samples.

**Conclusion:**

This study provides updated and cross-cultural data showing that dissociation is associated with an increase in PTSD symptoms over time, though its association with DSO symptoms is less clear. Assessment, prevention, and treatment of PTSD should take dissociative symptoms into consideration.

Post-traumatic stress disorder (PTSD) and dissociation are closely related, and both are common responses to trauma exposure, especially interpersonal and betrayal trauma [1–4]. In both ICD-11 and DSM-5-TR, dissociative disorders are placed directly after the section on trauma-related disorders, indicating that both groups of disorders are highly linked to trauma [5–7]. Patients with severe dissociative disorders report the highest rates of childhood abuse [8], and complex PTSD (CPTSD) has also been widely conceptualized as a response to chronic interpersonal trauma [9]. Dissociation is broadly defined as failures in the process of *integrating* one’s own biopsychosocial experiences (e.g., memories, behaviors, emotions, identities) [10, 11]. Therefore, dissociation can be understood as state in which intrapersonal experiences become disconnected from conscious awareness [12]. Although dissociative experiences can occur in non-traumatic contexts, the construct of dissociation remains most salient in the context of psychological trauma.

Trauma, while not easy to define [12], is generally described as an event in which “(an) individual’s ability to integrate his/her emotional experience is overwhelmed” [13]. Linguistically, the problem with failing to *integrate* one’s own experience essentially denotes dissociation, as dissociation represents the opposite of integration. Therefore, numerous scholars have consistently theorized that dissociation is a core construct central to understanding traumatization [3, 14]. From this perspective, PTSD has been theorized either as a dissociative disorder itself [15, 16], or as a condition inherently involving dissociation [17]. This conclusion follows from the cumulative theoretical and empirical evidence reviewed above. For example, according to the theory of structural dissociation [3], complex PTSD involves both primary dissociation (characterized by a division between apparently normal and emotional parts of the personality) and secondary dissociation (includes further fragmentation of emotional parts in response to chronic trauma) [15]. Theoretically speaking, PTSD symptoms arise because there are dissociated (unprocessed) emotions and memories. Some PTSD symptoms, such as re-experiencing, are also classified as dissociative (e.g., dissociative flashbacks) in DSM-5-TR. This theoretical perspective, while seen as valid for many, has also been challenged. This is described more below.

The dissociative subtype of PTSD is recognized in the DSM-5-TR, yet includes only two dimensions of dissociation: depersonalization and derealization. In the ICD-11, neither PTSD nor complex PTSD (CPTSD) includes dissociative symptoms as required diagnostic features, although an earlier formulation of CPTSD proposed dissociation as one of its core features [9, 18, 19]. Two recent studies, using different statistical methods, found that CPTSD can exist without significant levels of dissociation; only 42.3% of those with CPTSD exhibited dissociative symptoms [16, 17]. Therefore, recent research findings do not support the notion that PTSD or CPTSD is, per se, a dissociative disorder. This apparent incongruence may reflect differences between earlier theoretical conceptualizations, in which many of which pre-dated the ICD-11 and were based on descriptive or clinical models, and more recent latent-variable and empirical frameworks, such as those used to develop the ITQ.

Nevertheless, dissociation might still play an important role in the development and maintenance of PTSD symptoms. It has been proposed that dissociative symptoms might mediate the relationship between trauma and subsequent psychopathology (including PTSD symptoms) [5], with significant implications for prevention and early interventions. If a traumatized individual continues to enter or experience dissociation as a (maladaptive) coping mechanism, they have fewer opportunities to confront, process, accept, and integrate trauma-related memories and emotions, which may lead to the persistence of PTSD symptoms because detaching from distressing internal states may lead to interruptions of the natural processes of emotional engagement and cognitive integration that are central to trauma recovery. Over time, these experiences remain fragmented, encoded as isolated sensory or emotional traces rather than integrated recollections, which could sustain intrusive imagery and emotional numbing. However, there is a lack of studies on the bidirectional relationship between dissociation and CPTSD symptoms, especially since the launch of ICD-11. The ICD-11 identifies two primary clusters of trauma-related symptoms: namely, classical PTSD symptoms and disturbances in self-organization (DSO) symptoms. Two recent studies aimed to address this research gap, but the results were inconsistent. One study with a sample of 340 Chinese young adults found that baseline dissociative symptoms predicted overall CPTSD symptoms at 4-month follow-up, but not the other way around [20]. Another study, however, did not observe that CPTSD and dissociative symptoms predicted each other in two samples (*N* = 214 Chinese speakers and *N* = 301 English speakers) [21].

In this study, we aim to advance the understanding of the complex relationship between CPTSD and dissociative symptoms. We analyzed data from two culturally different samples and provide updated cross-national evidence regarding (1) the co-occurrence of PTSD/CPTSD and dissociative symptoms, and (2) their bidirectional relationship. While this study was exploratory in nature, we hypothesized that dissociative symptoms would predict both PTSD and DSO symptoms at a later time because dissociation is a barrier to trauma recovery. When a person continue to dissociate, they may not have enough opportunities to process and integrate the trauma-related memories and emotions, resulting in ongoing post-traumatic reactions including but not limited to re-experiencing, avoidance, negative self-concept, and emotional dysregulation, thus leading to subsequent PTSD and DSO symptoms.

Previous studies showed that there are cultural differences in terms of trauma responses, post-traumatic growth, and stress coping [22–24]. Therefore, it is important to replicate our results across culturally different samples and examine whether the results are valid across cultures. While the samples differ in cultural background, our focus is on cross-cultural replication rather than an explicit examination of cultural mechanisms; cultural considerations are addressed in the discussion section. Findings from the current study would have significant implications for the cross-national understanding of the role of dissociation in PTSD and CPTSD research.

## Methods

### Participants

This study analyzed data from an international female mental health survey project, which obtained ethics approval at Leshan Normal University. This is a collaborative project. In 2024, the research team recruited potential participants in clinics and affiliated community centers and through online social media advertising. Eligibility criteria included being female aged 18 or above, provide informed consent, possess the ability to read and write in English, receive any kind of mental health services in the past 12 months, and have not been officially diagnosed with reading disorders, or any cognitive or intellectual impairments. Participants were excluded if they failed to answer the embedded attention checking items (e.g., *4 + 3=?*). Participants with duplicate email addresses were also excluded from the analysis. Although no monetary incentives were given, participants were offered a free e-copy of a mental health self-help booklet as a token of gratitude for their time upon survey completion.

In total, the baseline survey included valid responses from 208 participants from the Western countries (i.e., United States, United Kingdom, and Canada) and 208 from the South Asian countries (i.e., Indonesia, Philippines, Singapore, and Malaysia). All participants were invited to complete a follow-up online survey approximately 6 months later. Previous longitudinal studies have demonstrated that PTSD symptoms often exhibit clinically meaningful changes after 6 months, including substantial spontaneous remission and reductions in symptom severity [25, 26]. Thus, the 6-month longitudinal observation period was selected to capture trajectories of PTSD/CPTSD in our samples and whether these symptoms would be associated with dissociation. The included sample for the present study comprised only of participants who provided valid data at both time points, resulting in a response rate of 48.6% in the Western samples and 26.6% in the South Asian sample. In each sample, participants completed the International Trauma Questionnaire (ITQ) to assess the PTSD and DSO symptoms and they also completed the Multiscale Dissociation Inventory (MDI) to assess dissociative symptoms at both time points.

### Sample characteristics

Sample 1 consisted of 101 participants from Western countries (57.4% North America/Canada, 42.6% the United Kingdom). Their ages ranged from 18 to 64. At baseline, 65.3% and 47.5% reported seeing a psychiatrist or a clinical psychologist, respectively, in the past 12 months. Participants who completed the follow-up survey (*N* = 101) and those who did not (*N* = 107) did not differ in any major variables (i.e., age, dissociation, PTSD, and DSO) at baseline.

Sample 2 consisted of 160 participants from South Asian countries (36.9% Malaysia, 30% the Philippines, 22.5% Indonesia, 10.6% Singapore). Their ages ranged from 18 to 45. At baseline, 53.1% and 48.1% reported seeing a psychiatrist or a clinical psychologist, respectively, in the past 12 months. Participants who completed the follow-up survey (*N* = 160) and those who did not (*N* = 442) did not differ in age and DSO symptoms at baseline, but those who did not complete the follow-up survey had higher levels of PTSD (M = 13.50 vs. M = 11.38, *p* <.001) and dissociative symptoms (M = 71.17 vs. M = 63.69, *p* =.002).

Detailed demographics are included in Table 1. No statistically significant differences were observed between the two samples at baseline in terms of key demographics, except for education level (see Table 1). The South Asian sample had a substantially higher proportion of participants with a bachelor’s degree (79.38%) compared with the Western sample (48.51%).

**Table 1:** Sample Characteristics

	Variable	All Count *N* (%)/mean (sd)	Western Count *n* (%)/mean (sd)	South Asian Count *n* (%)/mean (sd)	t/X^2^	*p*
Gender	Female	261 (100%)	101	160	−	−
Age	Age	26.34 (7.11)	26.04 (9.27)	26.53 (5.35)	− 0.485	0.629
Education	Bachelor’s Degree	122 (67.40%)	49 (48.51%)	127 (79.38%)	26.850	< 0.001
Employment/Occupation	Full Time employment	94 (36.02%)	27 (26.73%)	67 (41.88%)	6.161	0.013
Marital Status	Married or in a common-law partnership	53 (20.31%)	26 (25.74)	27 (16.88%)	3.009	0.083
Mental Health Service Utilization (Past Year)	Psychiatrist	151 (57.85%)	66 (65.35%)	85 (53.13%)	3.793	0.051
	Clinical psychologist	125 (47.89%)	48 (47.54%)	77 (48.13%)	0.009	0.925
Mental Health Measures	MDI	65.52 (26.09)	68.43 (27.77)	63.69 (24.88)	1.430	0.154
	PTSD	12.01 (6.93)	13.03 (7.09)	11.37 (6.77)	1.888	0.060
	DSO	14.64 (6.32)	14.86 (6.52)	14.51 (6.21)	0.441	0.659
	Probable ICD-11 PTSD or CPTSD	115 (44.06%)	48 (47.52%)	67 (41.88%)	0.802	0.371
Childhood trauma	BBTS	3.28 (2.50)	3.14 (2.54)	3.37 (2.48)	−0.723	0.470
	Any	227 (86.97%)	86 (85.15%)	141 (88.1%)	0.484	0.487

PTSD = post-traumatic stress disorder; DSO = disturbances in self-organization

### Measures

Participants completed the following self-report measures in English.

*Childhood trauma* was assessed using the 12-item Childhood Trauma Section of the Brief Betrayal Trauma Survey (BBTS) [27]. The BBTS includes items which ask about both interpersonal and non-interpersonal traumatic events. It has been found to be a reliable measure [28]. It ranged from 0 (no traumatic event) to 12 (all included traumatic events).

*CPTSD symptoms* were assessed using the International Trauma Questionnaire (ITQ), which is an 18-item measure of CPTSD symptoms (including PTSD and disturbances in self-organization [DSO] symptoms) as defined in ICD-11 [29, 30]. The ITQ can also be used to make suggestive presence of PTSD/CPTSD according to ICD-11 criteria – this method has been widely used in epidemiological studies [31, 32]. Total ITQ scores ranged from 0 to 24.

*Dissociative symptoms* were assessed using the Multiscale Dissociation Inventory (MDI), which is a 30-item measure of six different specific dissociative symptoms [33, 34]. The measure provides scores on six sub-domains, including Disengagement, Depersonalization, Derealization, Emotional Constriction/Numbing, Memory Disturbance and Identity Dissociation. The MDI has been validated across cultures in a recent study [35]. Since the MDI has been normed [36], a cutoff score of 67 is commonly used to indicate elevated (clinically significant) levels of dissociation [37]. Total scores ranges from 30 to 150.

### Data analysis

SPSS 22.0 was used for statistical analysis. First, we provided descriptive statistics. Independent sample t tests and chi-square analyses to explore potential differences between the two samples at baseline. Second, we reported the rates of co-occurrence of probable ICD-11 PTSD/CPTSD and dissociation in each sample. Third, we conducted hierarchical linear regression analyses to examine the bidirectional relationship between CPTSD symptoms and dissociative symptoms. For each analysis, we controlled for age, education level, childhood trauma, and T1 scores of the dependent variable in Step 1, and then entered the T1 scores of the predictor in Step 2. Because of multiple testing involved in the analyses, we further applied the Bonferroni correction and conservatively evaluated whether the results would be statistically significant at the level of 0.0083 (0.05/6).

## Results

### Co-occurrence

We calculated the co-occurrence rates of dissociation and probable PTSD/CPTSD at T1 in each sample.

In the Western sample, among participants with dissociation (MDI ≥ 67)(*n* = 47), 72.3% had probable PTSD (4.3%) or CPTSD (68.1%). Among participants with probable PTSD (*n* = 10), 20.0% had dissociation. Among participants with probable CPTSD (*n* = 38), 84.2% had dissociation.

In the South Asian sample, among participants with dissociation (MDI ≥ 67)(*n* = 60), 70.0% had probable PTSD (6.7%) or CPTSD (63.3%). Among participants with probable PTSD (*n* = 14), 28.6% had dissociation. Among participants with probable CPTSD (*n* = 53), 71.7% had dissociation.

### Could dissociative symptoms predict CPTSD symptoms?

Hierarchical linear regression showed that, after controlling for age, education, childhood trauma, and T1 PTSD symptoms, T1 dissociative symptoms predicted T2 PTSD symptoms in both Western (β = 0.241, *p* =.006) and South Asian (β = 0.246, *p* =.003) samples. The results remained statistically significant after applying the Bonferroni correction (*p* <.0083).

For DSO symptoms, T1 dissociative symptoms predicted T2 DSO symptoms in the South Asian sample (β = 0.231, *p* =.011) but not in the Western sample (β = 0.093, *p* =.311).

The results are reported in Tables 2 and 3.

**Table 2:** The relationship between complex PTSD and dissociative symptoms in the Western sample (*N* = 101)

Variables	T2 PTSD symptoms				T2 DSO symptoms				T2 Dissociative symptoms			
	Step 1		Step 2		Step 1		Step 2		Step 1		Step 2	
	β	*p*	β	*p*	β	*p*	β	*p*	β	*p*	β	*p*
Age	−0.065	0.407	−0.025	0.743	−0.065	0.37	−0.045	0.543	0.016	0.809	−0.003	0.958
Education level (Bachelor’s degree)	0.014	0.852	0.017	0.823	−0.013	0.859	−0.021	0.776	0.017	0.785	0.075	0.255
Childhood trauma	0.047	0.585	−0.009	0.92	0.043	0.558	0.017	0.829	0.006	0.929	−0.012	0.863
T1 Dissociative symptoms			0.241	0.006			0.093	0.311	0.811	< 0.001	0.698	< 0.001
T1 PTSD symptoms	0.681	< 0.001	0.578	< 0.001							0.065	0.404
T1 DSO symptoms					0.734	< 0.001	0.681	< 0.001			0.155	0.06
R^2^	0.489		0.528		0.564		0.569		0.655		0.673	
Adjust R^2^	0.467		0.503		0.546		0.546		0.652		0.652	
F	22.927***		21.239***		31.023***		25.036***		45.612***		32.295***	
ΔR^2^	0.489		0.039		0.564		0.005		0.655		0.018	
ΔF	22.927***		7.897**		31.023***		1.039		45.612***		2.607	

** *p* <.01 *** *p* <.001; PTSD = post-traumatic stress disorder; DSO = disturbances in self-organization

**Table 3:** The relationship between complex PTSD and dissociative symptoms in the South Asian sample (*N* = 160)

Variables	T2 PTSD symptoms				T2 DSO symptoms				T2 Dissociative symptoms			
	Step 1		Step 2		Step 1		Step 2		Step 1		Step 2	
	β	*p*	β	*p*	β	*p*	β	*p*	β	*p*	β	*p*
Age	−0.062	0.368	−0.032	0.637	−0.188	0.008	−0.162	0.021	−0.15	0.017	−0.14	0.026
Education level (Bachelor’s degree)	0.079	0.247	0.071	0.285	0.105	0.128	0.103	0.128	0.079	0.195	0.073	0.232
Childhood trauma	0.049	0.5	0.008	0.914	−0.002	0.978	−0.057	0.413	−0.044	0.489	−0.063	0.337
T1 Dissociative symptoms			0.246	0.003			0.31	0.011	0.681	< 0.001	0.604	< 0.001
T1 PTSD symptoms	0.576	< 0.001	0.448	< 0.001							0.068	0.404
T1 DSO symptoms					0.536	< 0.001	0.406	< 0.001			0.068	0.401
R^2^	0.378		0.413		0.36		0.387		0.505		0.512	
Adjust R^2^	0.362		0.394		0.344		0.367		0.492		0.493	
F	23.564***		21.649***		21.839***		19.443***		39.530***		26.750***	
ΔR^2^	0.378		0.035		0.36		0.027		0.505		0.007	
ΔF	23.564***		9.079**		21.839***		6.666**		39.530***		1.094	

** *p* <.01 *** *p* <.001; PTSD = post-traumatic stress disorder; DSO = disturbances in self-organization

We conducted the same analyses again without controlling for childhood trauma, and the results remained the same.

### Could CPTSD symptoms predict dissociative symptoms?

When the same models were conducted, we found that T1 PTSD and DSO symptoms did not predict T2 dissociative symptoms in both samples (see Tables 2 and 3).

We also conducted the same analyses again without controlling for childhood trauma, and the results remained the same.

## Discussion

PTSD and CPTSD have long been conceptualized as involving dissociation and it has been theorized that dissociation might lead to the development or maintenance of post-traumatic symptoms [3, 14]. Nevertheless, recent studies found that only a subgroup of people with CPTSD had clinically significant dissociative symptoms [16, 17]. Most prior studies use single-country samples and rely on cross-sectional designs. In this new study, we recruited two culturally distinct adult samples (Western and South Asian) and followed them over six months, enabling direct cross-cultural replication. Across the Western and South Asian samples, we found that 71.7% to 84.2% of participants with probable CPTSD exhibited co-occurring dissociative symptoms, while 70.0% to 72.3% of participants with dissociative symptoms had probable PTSD or CPTSD. Dissociative symptoms were much less common in participants with probable PTSD (20.0% to 28.6%). In addition, we found that dissociative symptoms predicted subsequent levels of classical PTSD symptoms across the two samples (β = 0.241 to 246), and the results remained statistically significant after applying the Bonferroni correction (*p* <.0083). The predictive role of dissociative symptoms on DSO symptoms, however, were not consistent, as it was only observed in the South Asian sample (β = 0.231, *p* =.011). Neither PTSD nor DSO symptoms predicted dissociative symptoms in both samples.

The results contribute to the limited literature on the co-occurrence of [16, 17] and bidirectional relationship [18, 20] between CPTSD and dissociative symptoms. In a previous study, only 42.3% of people with CPTSD exhibited dissociative symptoms. Yet, in the present study, we found that 71.7% to 84.2% of participants with probable CPTSD exhibited co-occurring dissociative symptoms. The difference might be due to the use of different dissociation measures (The DES-Taxon versus the MDI). Another possibility is that the samples were not representative enough. Moreover, our co-occurrence analyses showed that dissociation is more related to CPTSD/DSO instead of classical PTSD. However, our regression analyses showed that dissociation predicted subsequent PTSD but not DSO symptoms. Such inconsistent findings in fact point to the need for further studies in the future. While subgroup analyses would not be feasible due to the small sample sizes in the present studies, future studies should examine whether the predictive role of dissociation would be consistent in both (C)PTSD and non-(C)PTSD cases. Thus, more research on the prevalence of dissociative symptoms and disorders among people with PTSD and CPTSD is necessary for us to better understand the role and clinical relevance of dissociation in CPTSD. Nevertheless, our findings suggest that, although not all cases of PTSD and CPTSD involve clinically significant dissociative symptoms, dissociation predicts PTSD symptoms over time, though its relationship with DSO symptoms is less clear and consistent. This also supports the idea that PTSD, DSO, and dissociation are related but distinct trauma responses, and dissociative symptoms might be crucial in the maintenance of PTSD symptoms. The results are in line with a recent clinical study showing that dissociation predicted poorer response to PTSD treatment [38]. It implies that prevention and early treatment of PTSD should take dissociation into account, and dissociation-specific interventions might be particularly useful for PTSD symptoms. As two recent randomized controlled trials showed that scalable online psychoeducation programs effectively supports individuals with dissociative symptoms [39, 40], these interventions may also have the potential to become a public health strategy to prevent PTSD in trauma-exposed populations, although further evaluation studies are necessary.

With regards to the weak prospective influence of PTSD and DSO on later dissociation, it points to a deeper developmental substrate. Dissociation often reflects patterns laid down long before adult symptom expression, particularly in contexts where early caregiving is inconsistent, threatening, or overwhelming [41, 42]. Our findings are consistent with the view that dissociative tendencies function less as downstream consequences of current PTSD/CPTSD severity and more as enduring adaptations shaped early in life. It may also be highly context-dependent. Childhood cumulative trauma was consistently associated with PTSD, DSO, and dissociative symptoms [43–46], emphasizing its broad developmental influence across these domains. Early adversity contributes to vulnerability for all three forms of trauma-related symptoms, rather than selectively shaping only one pathway. Our findings reinforce longstanding evidence that childhood trauma establishes enduring risk for both post-traumatic symptom clusters and dissociative tendencies into adulthood. Dissociation may be more strongly associated with the trajectory of PTSD symptoms instead of DSO symptoms, as revealed by our findings, probably because PTSD and DSO symptoms involve different etiological mechanisms.

### Clinical implications

In the context of therapeutic practices, dissociation undermines several mechanisms that PTSD therapies rely on. For example, it disrupts sustained attention, limits emotional engagement with traumatic material, fragments autobiographical memory, and could blunts access to coherent affective signals [47–49]. These are all capacities required for effective exposure and integration. When dissociation is active, patients often cannot stay within the therapeutic window long enough to process trauma cues or consolidate corrective learning. It may heighten avoidance and complicate moment-to-moment arousal monitoring. Our findings suggest that change in PTSD symptoms should be closely monitored in outpatients who report dissociative symptoms.

### Limitations

This study offers updated, cross-cultural data to enhance our understanding of the complex relationship between PTSD, DSO, and dissociation. Its strengths include the use of longitudinal data, replication across two culturally diverse samples, application of the Bonferroni correction, and the use of well-validated assessment tools. We in fact provide evidence showing that dissociative symptoms predict an increase in PTSD symptoms across cultures. However, this study also has several limitations. First, we relied on self-report data and did not conduct diagnostic interviews to confirm the clinical status of the participants, although self-report screening tools are found to be reliable and valid and have good agreement with diagnostic interviews for dissociation [50–52]. Second, our samples were not representative of the clinical population, and self-selection bias might have taken place during participant recruitment. Third, given the relatively small sample sizes, we only used the MDI total scores and were unable to treat dissociation as a multidimensional construct [33, 34, 53]. Fourth, given that only some participants met the diagnostic requirements for probable PTSD/CPTSD in our samples, the results of the regression analyses are not specific to patients with those diagnoses nor those with clinically significant levels of dissociative symptoms. Further studies using samples with confirmed diagnoses are needed. In addition, future studies should employ diagnostic interviews and use a more representative sample to replicate our findings. While further research is needed, prevention and treatment of PTSD should start to pay more attention to dissociative symptoms.

It is important to note that the MDI captures a broad range of dissociative experiences, including non-clinical forms (e.g., absorption). This wider scope may partly explain the higher comorbidity rates observed here compared with studies using measures focused more narrowly on structural or clinically severe dissociation. Because the DSM-5 dissociative subtype is defined specifically by depersonalization and derealization, analyses isolating the corresponding MDI items could clarify whether this subtype maps only onto PTSD or also extends to CPTSD and DSO. Although such item-level analyses were beyond the scope of the present study, they represent a useful direction for future work.

Moreover, the English-language requirement also introduces additional self-selection. In both settings, English proficiency often corresponds to greater educational access and, in some cases, socioeconomic advantage, likely excluding individuals with lower literacy or more limited schooling. This, combined with the broader demographic profile of our participants, primarily young adults (mean age = 26), many with childhood trauma histories and recent treatment involvement, and with the South Asian sample largely college educated, limits representativeness. Therefore, findings may not fully generalize to older adults, those without early adversity, individuals not engaged in treatment, or populations with lower educational access. Our results point to the value of treating dissociation as its own clinical process rather than a secondary feature of PTSD or CPTSD. Because dissociative symptoms predicted later PTSD but were not meaningfully shaped by changes in PTSD or DSO, dissociation may represent a more stable vulnerability that can interfere with core components of trauma-focused therapy.

Overall, the findings indicate that dissociation is associated with an increase in PTSD symptoms over time across two culturally distinct samples, while its link to DSO appears more context-dependent and less clear and consistent. The limited impact of PTSD/DSO on later dissociation suggests that dissociative tendencies may have deeper developmental roots and remain relatively stable into adulthood. We conclude that recognizing dissociation as a distinct mechanism, rather than a downstream effect of PTSD, may strengthen assessment and intervention efforts, particularly in cross-cultural research and clinical practice.

## Data Availability

Data that support the findings of this study are available from the corresponding authors upon reasonable request.
